# Genetic tools to study juvenile hormone action in *Drosophila*

**DOI:** 10.1038/s41598-017-02264-4

**Published:** 2017-05-18

**Authors:** A. A. Baumann, M. J. Texada, H. M. Chen, J. N. Etheredge, D. L. Miller, S. Picard, R. Warner, J. W. Truman, L. M. Riddiford

**Affiliations:** 1 Howard Hughes Medical Institute, Janelia Research Campus, Ashburn, VA 21047 USA; 2 University of Tennessee, College of Veterinary Medicine, Knoxville, TN 37996 USA; 3 National Institute of Neurological Disease and Stroke, NIH, Bethesda, MD 20892 USA; 4 Friday Harbor Laboratories, University of Washington, Friday Harbor, WA 98250 USA

## Abstract

The insect juvenile hormone receptor is a basic helix-loop-helix (bHLH), Per-Arnt-Sim (PAS) domain protein, a novel type of hormone receptor. In higher flies like *Drosophila*, the ancestral receptor *germ cell-expressed* (*gce*) gene has duplicated to yield the paralog *Methoprene-tolerant* (*Met*). These paralogous receptors share redundant function during development but play unique roles in adults. Some aspects of JH function apparently require one receptor or the other. To provide a foundation for studying JH receptor function, we have recapitulated endogenous JH receptor expression with single cell resolution. Using Bacteria Artificial Chromosome (BAC) recombineering and a transgenic knock-in, we have generated a spatiotemporal expressional atlas of *Met* and *gce* throughout development. We demonstrate JH receptor expression in known JH target tissues, in which temporal expression corresponds with periods of hormone sensitivity. Larval expression largely supports the notion of functional redundancy. Furthermore, we provide the neuroanatomical distribution of JH receptors in both the larval and adult central nervous system, which will serve as a platform for future studies regarding JH action on insect behavior.

## Introduction

Juvenile hormone (JH) drives various aspects of insect physiology. A well-documented role for this hormone is to prevent early metamorphosis. In most insects, JH prevents early induction of key metamorphosis-initiating genes. As larvae gain competence to undergo metamorphosis^1^, the JH titer drops, reducing expression of the antimetamorphic, JH response gene Kr-h1, which lifts suppression on pupal or adult developmental programs^2, 3^. This aspect of JH activity has promoted the synthesis of batteries of compounds employed to combat pest insects. These JH analog (JHA) insecticides have been used in various formulations to combat medical and agricultural pests^4, 5^ since their exogenous application can disrupt normal development. Methoprene, a JHA that shares structural similarity with endogenous JH, has proven valuable as both a research tool and as a key component of flea and tick preventative products for companion animals.

Unlike beetles and moths, in which JHAs can induce supernumerary larval instars, JH exposure does not override metamorphosis in flies^6^, which instead die as pharate adults that fail to eclose from the puparium. Furthermore, the best candidate for a *Drosophila* JH receptor, the bHLH PAS protein Met, was demonstrated to be encoded by a nonvital gene^7^. Thus, despite its utility as a powerful genetic model, progress toward understanding JH signaling in *Drosophila* has been frustratingly slow. However, Konopova and Jindra^8^ demonstrated that suppressing the presumed *Met* ortholog in *Tribolium* using RNAi induced developmental phenotypes that were typical of a loss of JH signaling; specifically, *TcMet*-deficient animals experienced precocious metamorphosis. Sequence analysis demonstrated that *TcMet* is ancestral to another *Drosophila* bHLH PAS gene called *gce* ^9^, and that *DmMet* arose during dipteran evolution^10, 11^. *Met* ^*27*^ *gce* ^*2*.*5k*^ double null larvae suffer developmental arrest during pupariation^12^, which phenocopies the effects of the loss of JH achieved through genetic ablation of the JH-producing gland, the corpus allatum (CA) (allatectomy, CAX;^13^). Unlike CAX larvae^14^, *Met* ^*27*^ *gce* ^*2*.*5k*^ larvae cannot be rescued by supplying exogenous JHA^12^. However, they can be rescued with a single ectopic copy of either gene, demonstrating redundancy. Recent work has demonstrated that Met and Gce proteins each bind JH with high affinity and that this binding is eliminated when the ligand-binding pocket is mutated^15^. As *bona fide* paralogous JH receptors (JHR), these proteins represent a novel class of metazoan hormone receptor^16^.

Paralog-specific function for each JH receptor has primarily been described in adults^17, 18^. In addition to its developmental and reproductive roles, JH signaling also influences insect behaviors, including courtship^17, 19^, aggression^20^, and the onset of foraging behavior in honey bees^21^. While studies have demonstrated how JH acts during nervous system development^14, 22^, we understand virtually nothing about how JH influences the neuronal circuits underlying various insect behaviors.

To provide a framework for addressing the tissue- and cell-specific action of JH, we determined the spatiotemporal expression of JH receptors through *Drosophila* development using a series of genetic tools. Specifically, we recapitulated endogenous *Met* and *gce* expression in both larval and adult *Drosophila melanogaster* using both recombineered BAC drivers and a viral T2A peptide-mediated transgenic knock-in to drive the expression of a variety of fluorescent reporter constructs. Our results suggest that *Met* and *gce* share considerable expressional overlap in larval *Drosophila*, supporting the notion of functional redundancy during preadult development^12^. There were a few key exceptions to this finding, in which one receptor was expressed at substantially higher levels than, or in exclusion to, its paralog. Furthermore, we demonstrate JH receptor expression in known JH target tissues, the temporal dynamics of which are consistent with periods of hormone sensitivity. In both larvae and adults, paralog-specific expression was evident predominantly in the central nervous system (CNS), suggesting that they have distinct contributions to JH-regulated behavior and CNS development. Here, we provide a neuroanatomical atlas of JHR expression, which will ultimately facilitate future studies regarding JH action in CNS development and function.

## Results

### BAC Validation

**Figure 1:**
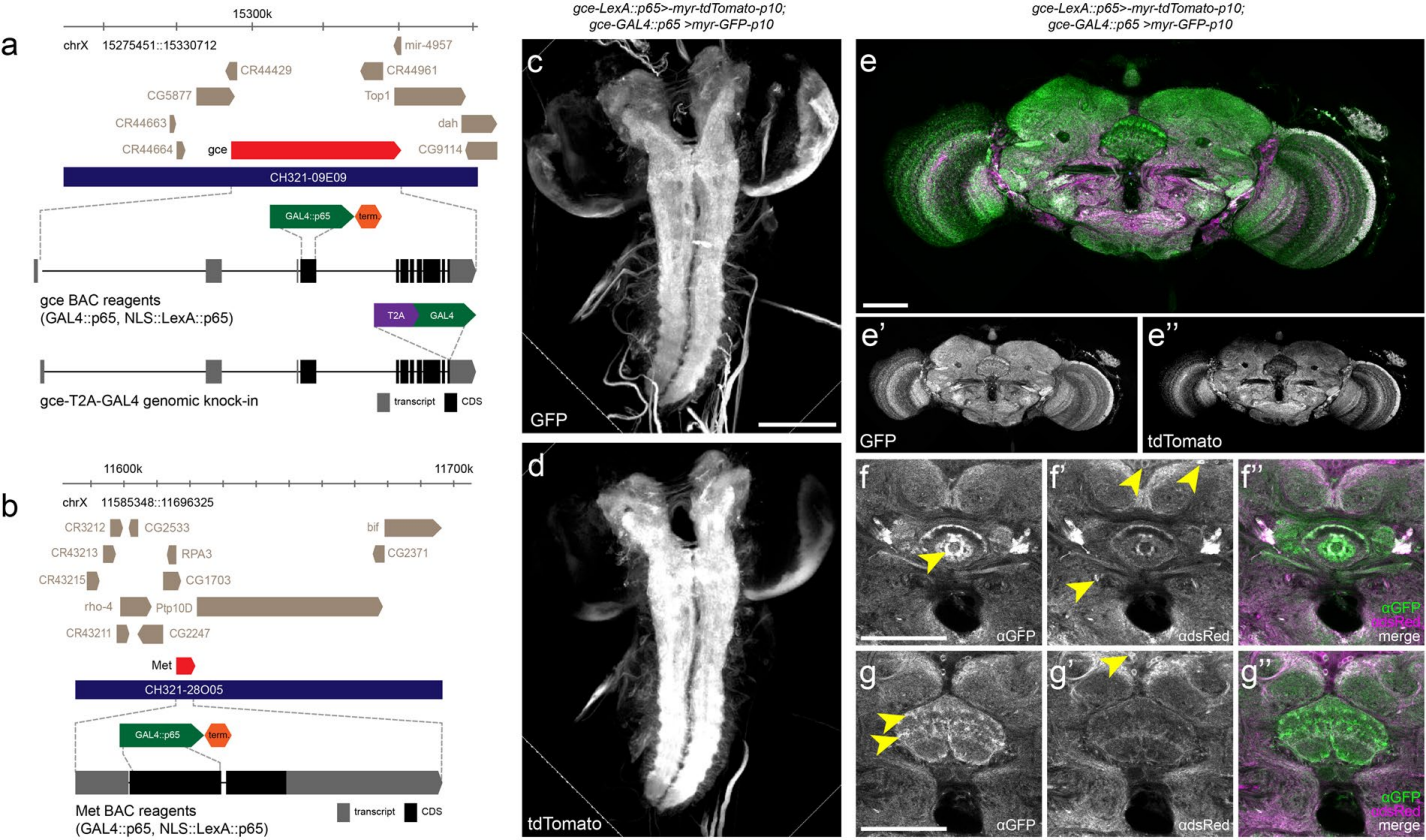
Construction of BAC and T2A-GAL4 reagents and validation of GAL4 and LexA iterations through co-expression. (**a**) The region of the X chromosome that contains the corresponding sequence contained on the native *gce* BAC, CH321-09E09. The *gce* locus is shown in red, while surrounding genes are shown in tan, showing proper syntenic organization according to the FlyBase GBrowse function (www.FlyBase.net). The overall segment corresponding to the sequence contained on the BAC is shown in navy, and under this are representative cartoons for the *gce-GAL4::p65* BAC and *gce-T2A-GAL4* constructs. (**b**) The *Met-GAL4::p65* BAC was constructed similarly. (**c**–**g”**) Co-expression experiments reveal the degree of reporter overlap when two independent reporters with similar architecture were driven by *gce-GAL4::p65* and *g*ce*-LexA::p65* in the same line. (**c**) Expression pattern of *UAS-IVS-myr-GFP-p10* and (**d**) *LexAop-IVS-myr-tdTomato-p10* in the CNS of wandering third instar larvae. (**e**) Coexpression in the adult CNS, and (**e’**) GFP or (**e”**) tdTomato channel only. Coexpression across the ellipsoid body (**f**–**f”**) and the fan-shaped body (**g**–**g”**) of the adult central complex. Yellow arrowheads indicate cells that show qualitatively higher expression under the particular driver-reporter combination, (GAL4/UAS or LexA/LexAop) relative to the other. GFP (green or white), α-dsRed (magenta).

**Figure 2:**
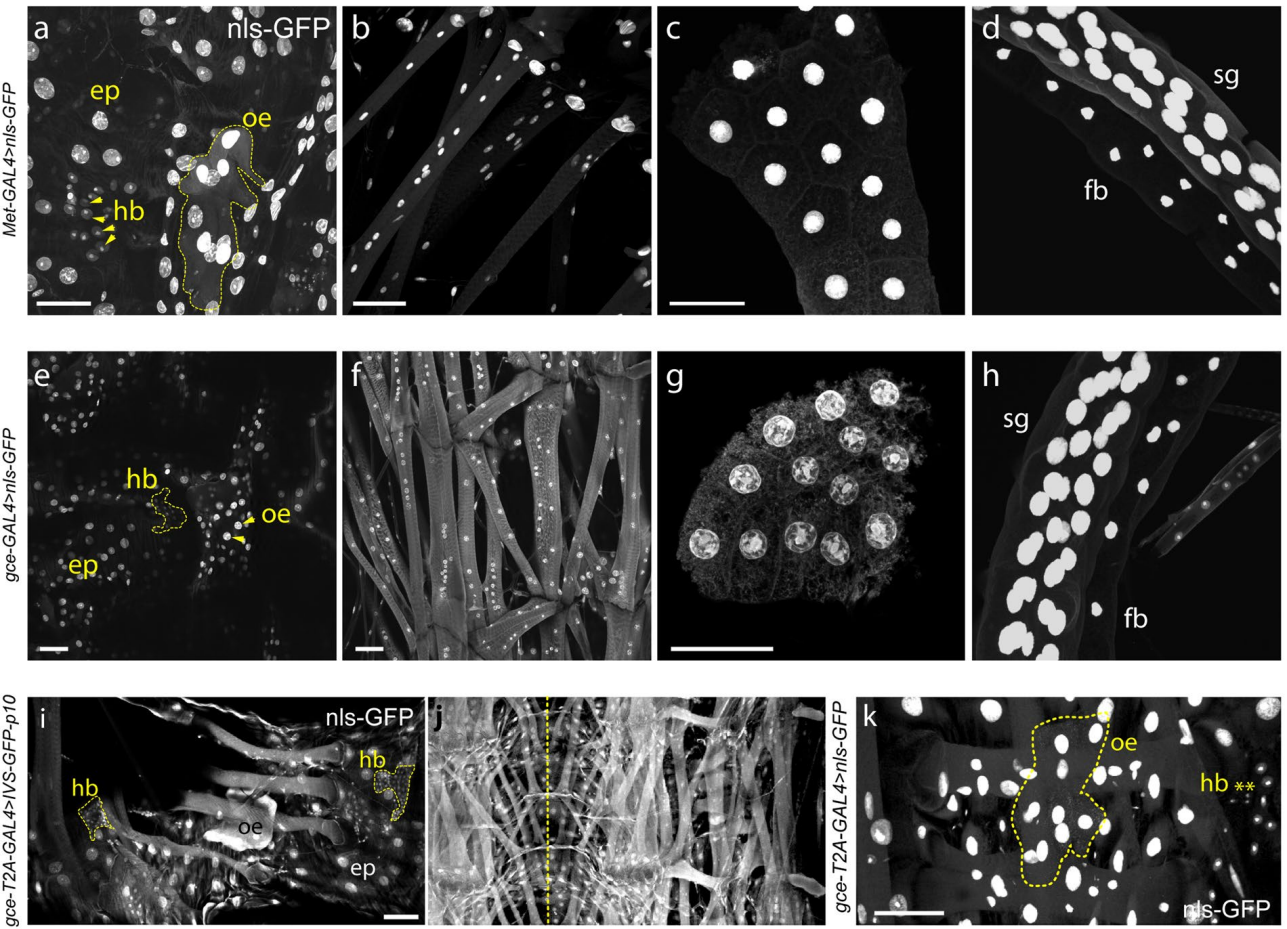
Confocal scans of larval filets reveal JHR expression in 3^rd^ instar larval body wall tissues, including epidermis, muscle, and fat body. The expression of the *Met-GAL4::p65* and *gce-GAL4::p65* constructs was visualized using the nuclear reporter Stinger-GFP in (**a**–**h**). The *gce-T2A-GAL4* construct was visualized by this reporter (**k**) or by the *10xUAS-IVS-p10-GFP* reporter (**i**,**j**). The patterns seen will be referred to as those of Met and Gce in the descriptions below. (**a**–**d**) Met expression in: (**a**) Large polyploid larval epidermal cells, diploid histoblasts (hb), and oenocytes (oe; dotted yellow outline) at the wandering stage, (**b**) larval muscles, (**c**) larval fat body, and (**d**) larval salivary gland (sg) and associated fat body (fb). (**e-h**) Gce expression in (**e**) epidermis, histoblasts, and oenocytes, (**f**) larval muscles (**g**) fat body cells, and (**h**) larval salivary glands (sg), shown with associated fat body (fb). (**i**) *gce-T2A-GAL4* > *10xUAS-IVS-GFP-p10* showed expression in histoblasts, epidermis, oenocytes, and larval muscles (m). (**j**) Gce expression was evident in all larval muscles (yellow dotted line: midline). (**k**) Close-up of *gce-T2A-GAL4* > *nls-GFP* expression in larval oenocytes (dotted line) and histoblasts (hb, **). Scale bars, 50 µM.

In this study we recapitulated endogenous JHR expression using transgenic *D*. *melanogaster* carrying either (1) one of several BACs comprised of a *gce* or *Met* coding sequence, interrupted by a GAL4::p65 or LexA::p65 driver fragment replacing the first exon, and flanked bilaterally by 40 kb of genomic DNA (Fig. 1a,b), or (2) a T2A-GAL4 fusion construct inserted in frame into the native *gce* genomic locus (Fig. 1a). These constructs were used to drive the expression of a number of fluorescent reporter constructs. We used several experimental approaches to validate the reported expression patterns.

First, we generated genomic rescue stocks containing the native BAC clones for *Met* or *gce*, which lacked a driver cassette. We crossed these lines *to Met* ^*27*^ *gce* ^*2*.*5k*^/*FM7c* females and scored the survival of progeny. *Met* ^27^ *gce* ^*2*.*5k*^ animals die early in pupal development, but can be rescued to adulthood with an ectopic *Met* or *gce* transgene^12^. Crosses of *Met* ^*27*^ *gce* ^*2*.*5k*^/*FM7c* females to *w* ^*1118*^ control males resulted in only FM7c/Y males, and their vials showed 25.9% pupal mortality (n = 194), accounting for *wvMet* ^*27*^, *gce* ^*2*.*5k*^/*Y* males that did not complete metamorphosis. Males carrying the native *gce* BAC eclosed at levels comparable with balancer sibs, indicating complete rescue (n = 174). By contrast, transformants carrying the native *Met* BACs rescued only 12% of *Met* ^*27*^ *gce* ^*2*.*5k*^ double mutants (n = 164) and the rescued males showed mosaic *w* ^+^ expression in their compound eyes. Therefore, while we were satisfied that the native *gce* BAC contained sufficient regulatory sequence to direct expression of functional *gce*, the native *Met* construct either could not restore JH signaling or contained genes which, when duplicated in the genome via ectopic expression, were incompatible with the JH receptor mutant background.

To determine whether *Met-GAL4::p65* could rescue *Met* phenotypes, we crossed the *Met-GAL4::p65* reagent into the *Met* ^*27*^ mutant line, but this stock was fragile and could not be maintained. We elected to sequence the genome of the *Met-GAL4::p65* reagent to evaluate the integrity of the construct. We discovered that the BAC contained small deletions in a 5′ gene, CG1703 (see Fig. 1b for position of CG1703). However, adjacent sequence corresponding to that contained in either the St-H fragment^23^ or the p[EN71] genomic rescue construct^24^ was intact. We therefore proceeded under the assumption that the *Met* expression reported using this BAC might represent the endogenous pattern.

Since we successfully generated multiple *gce* drivers, we compared the expression patterns reported among these various iterations. We compared *gce-LexA::p65* and *gce*-*GAL4*::*p65* directly in the same line: *gce*-*LexA*::*p65* (*attP40*) > *13xLexAop2*-*IVS*-*myr*-*tdTomato-p10* (*attP40*); *gce*-*Gal4*::*p65* (*VK00033*) > *10xUAS-IVS-myr-GFP-p10* (*attP2*). Confocal analysis revealed considerable overlap between *LexA*::*p65* and *GAL4*::*p65* reagents, both in adults and larvae (Fig. 1c–g”). Observed differences in spatial partitioning or expression strength of GFP reporters driven by the GAL4 and LexA BAC constructs were likely due to a combination of position (chromatin) effects, driver strength, and reporter architecture, as previously reported^25, 26^. In contrast, there were substantial differences in BAC- and T2A-reported GFP CNS expression for the *gce* gene, discussed below.

### Larval expression

**Figure 3:**
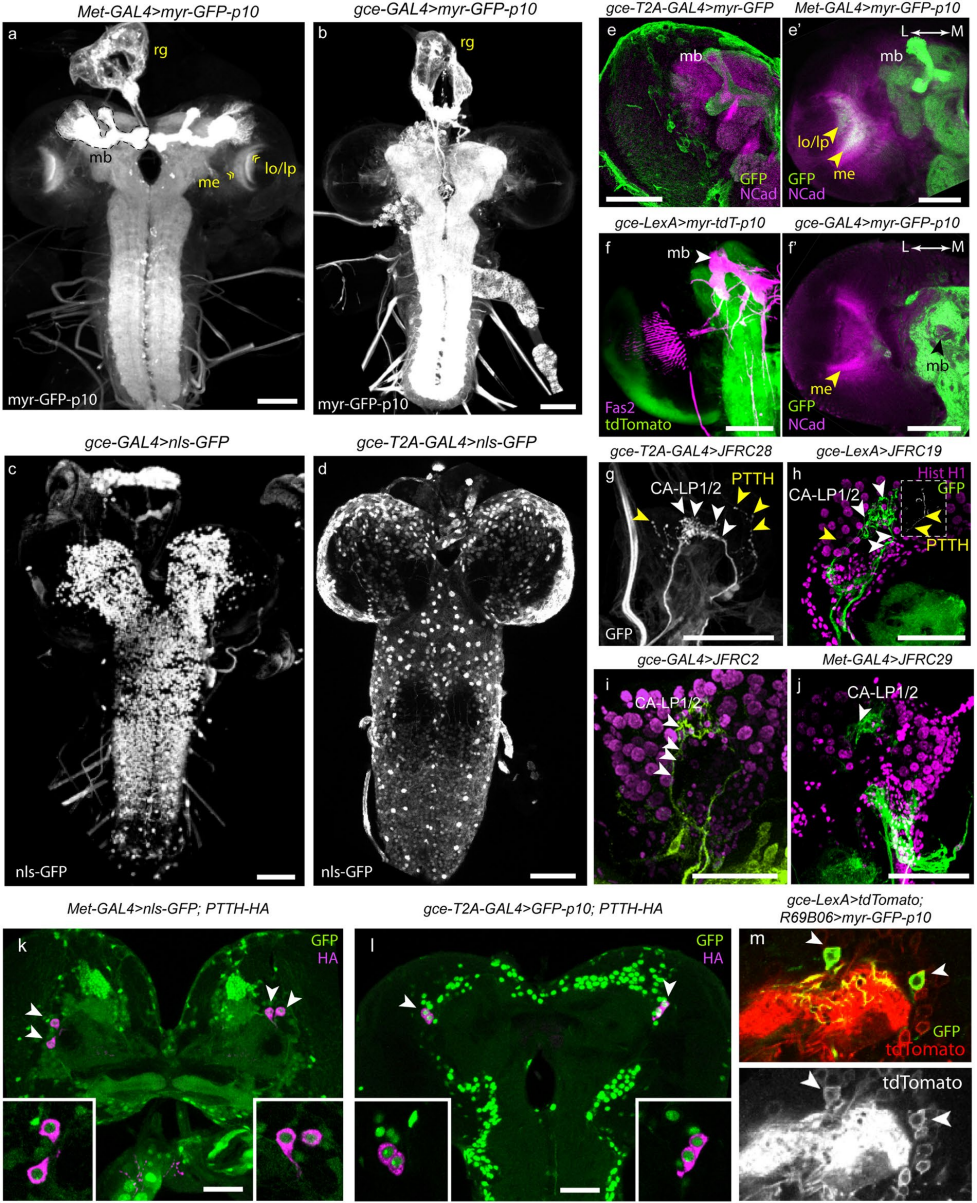
Confocal scans of JHR expression across the larval central nervous system, including neuronal expression in the ring gland. (**a**) *Met-GAL4::p65* > *myr-GFP-p10* expression across the larval CNS (wandering stage). Strong GFP signal was evident in the mushroom bodies (mb), one of which is outlined. The developing medulla (me) and lobula/lobula plate (lo/lp) neuropils are indicated by yellow chevrons. The ring gland (rg) is shown above the larval brain lobes. (**b**) *gce-GAL4::p65* > *myr-GFP-p10* expression across the larval CNS shows some restricted GFP signal in the larval mushroom bodies and no GFP in the developing optic lobe neuropils. *gce-GAL4::p65* (**c**,**f’**) and *gce-T2A-GAL4* (**d**,**e**) exhibited markedly different expression patterns in the larval CNS. Notably, the T2A reagent showed strong mushroom body (mb) and surface glia (sg) (yellow chevrons) expression (**e**); *gce*-LexA::p65 showed no mb expression (**f**), and *gce*-GAL4::p65 GFP expression in the mushroom bodies was substantially weaker than in the knock-in (**f’**). Anti-GFP antibody (green or white), αN-cadherin (magenta), Fas2 (magenta: **f**). Key ring gland neurons are identified as expressing Met/Gce using a variety of driver and reporter combinations. (**g**) *gce-T2A-GAL4* > *GFP-p10* expression in the larval ring gland (wandering stage) shows GFP in the CA-LP1/2 cells that innervate the corpus allatum (white arrowheads), as well as moderate expression in the PTTH cells (yellow arrowheads). (**h**) *gce-LexA::p65* > *myr-GFP*, (**i**) *gce-GAL4::p65* > *mCD8-GFP*, and (**j**) *Met-GAL4::p65* > *myr-GFP-p10* expression in CA-LP1/2 and PTTH neurons. In each case, CA-LP1/2 neurons are indicated with white arrowheads, while PTTH cells are indicated with yellow arrowheads. Insets in (**h**–**j**) serve to resolve PTTH expression. *Met* > nls-GFP (**k**) and *gce* > GFP (**l**) expression colocalizes with PTTH-HA in PTTH cell bodies; (white arrowheads and insets). (**m**) Colocalization of *R69B06* > *myr-GFP-p10* expression and *gce-LexA* > *tdTomato* in CA-LP1 and CA-LP2 cells (white arrowheads). All scale bars, 50 µM. GFP (green or white), αHistone H1 (magenta), αdsRed (red).

*Met-GAL4::p65* and *gce-GAL4::p65* or *gce-T2A-GAL4* each exhibited widespread tissue expression during larval growth including in epidermis, fat body, muscles, salivary glands (Fig. 2) and the central nervous system (CNS; Fig. 3). As in most other larval tissues, JHR expression was widespread in the larval CNS (Fig. 3a–d). One significant distinguishing characteristic was that *Met* > *myr-GFP-p10* showed strong expression in the mushroom bodies (mb) (Fig. 3a,e’), while *gce* > *myr-GFP-p10* showed moderate mb expression (Fig. 3b,f’), and *gce-LexA* > *myr-tdTomato-p10* showed none (Fig. 3f). In contrast to the BAC construction, the *gce* knock-in, *gce-T2A-GAL4*, showed obvious expression in the mushroom body (Fig. 3e). *Met* > *myr-GFP-p10* showed broad expression in the developing optic lobe, including the nascent lobula and medulla neuropils in WL3 stage larvae (Fig. 3a,e’), whereas *gce* neuronal expression using all three *gce* constructs and different reporters was essentially absent from the developing optic lobes (Fig. 3b–e,f and f’), but was evident in surface glia (Fig. 3d,e,f and f’). Both receptors were present in key peptidergic neurons whose axons project into the ring gland, including the prothoracicotropic hormone (PTTH) neurons that stimulate ecdysteroid production by the prothoracic glands (PG) (Fig. 3g,h,k and l) and the CA-LP1 and −2 neurons whose axons terminate in the corpus allatum (see Fig. 3g–j and m).

**Figure 4:**
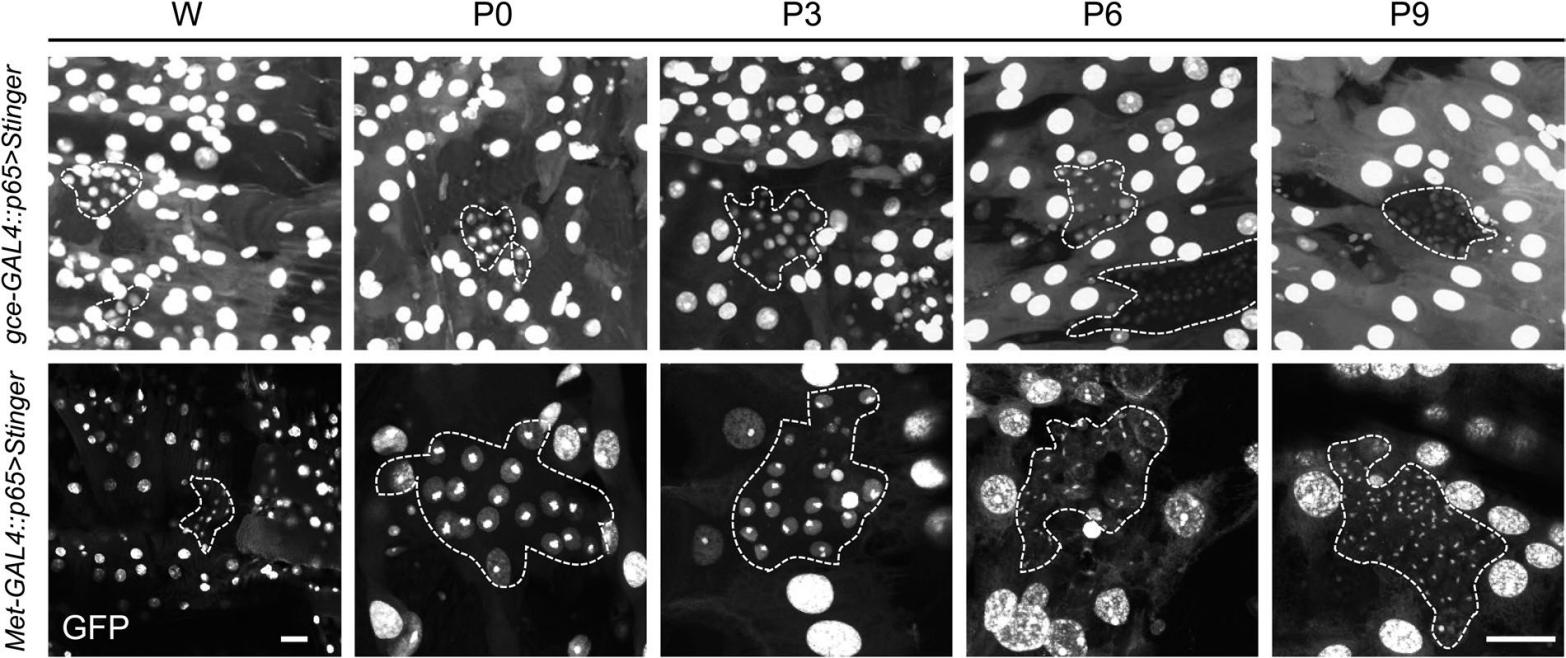
Developmental time course of JHR expression in histoblasts as shown by the Stinger nuclear reporter (*UAS-nls-eGFP*) for either *Met-GAL4::p65* or *gce-GAL4::p65*. **Top row**: *gce* expression from wandering through 9 hours after pupariation. **Bottom row**: *Met* expression through the same time period. The diploid histoblasts nests are outlined by the dashed lines, and are surrounded by polyploid larval epidermis and muscle nuclei, which are also GFP-positive. W: wandering; P0: white puparium stage; P(n): hours after pupariation. Scale bars, 50 µM.

In wild type flies, JH I or JH III application during the sensitive period severely disrupts adult abdominal differentiation by suppressing the proliferation and spreading of the abdominal histoblasts that normally give rise to the adult abdominal epidermis^27, 28^. *Met* mutants are refractory to this effect of JH treatment^29^. We detected both JHRs in wandering stage larva (WL3) histoblasts, with expression persisting through pupariation, and then tapering off by 9 h APF (Fig. 4).

### Imaginal discs

**Figure 5:**
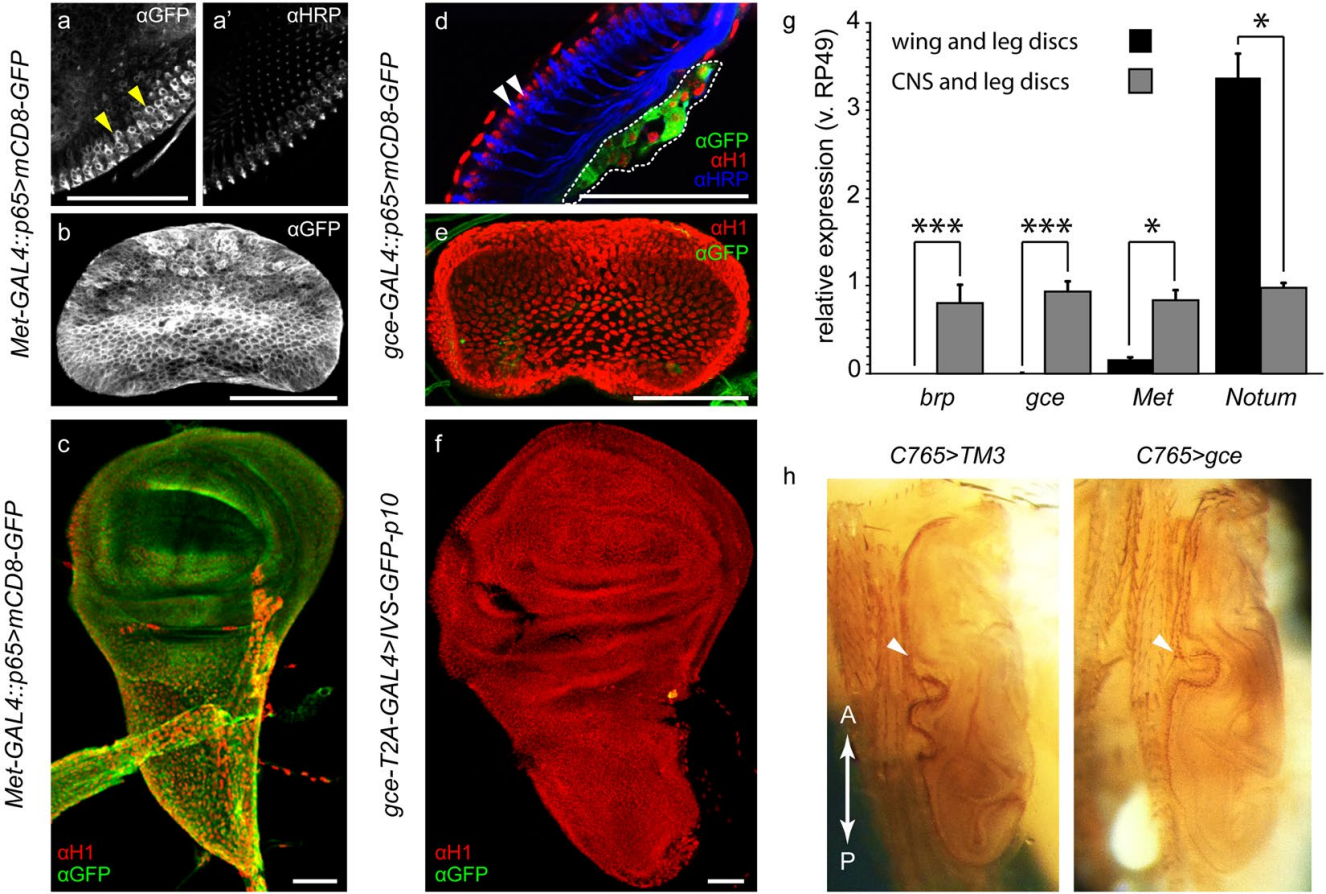
JHR expression in larval imaginal discs. (**a**) *Met-GAL4::p65* > *mCD8-GFP* expression in the eye disc, showing *Met* expression in the developing photoreceptors (yellow arrowheads), which show αHRP staining (**a’**). mCD8-GFP also shows *Met* expression globally in the genital disc (**b**) and wing disc (**c**) of wandering L3 animals. GFP expression was not detected in the eye (**d**) (αHRP staining is indicated by white arrowheads), genital (**e**) or wing discs (**f**) of wandering L3 animals, using either the *gce-GAL4::p65* or *gce-T2A-GAL4* drivers. The intensely-staining GFP-positive cells outlined by the white dotted line in (**d**) are adepithelial cells that adhere to the underside of the eye disc. (**g**) Quantitative PCR analysis of *Met* and *gce* expression in wing/leg discs vs. expression in CNS+ attached leg disc samples. *brp* serves as a CNS-specific control gene. *Notum* is enriched in wing discs relative to the CNS. Unpaired t-test *p < 0.05, ***p < 0.005; *n* = 135 for each gene: 3 technical replicates, each consisting of 3 biological replicates using material extracted from 15 animals. Error bars: SEM. (**h**) Normal development of the adult wing in animals fed 1 ppm dietary pyriproxyfen. Left: TM3 balancer sib control fly. Right: *C765* > *gce* fly expressing ectopic *gce* in the wing disc. Arrowheads indicate the anterior margin of the adult wing. Scale bars, 50 µM.

*Met-GAL4::p65* was expressed in wing, eye, leg, and genital discs (Fig. 5a–c and data not shown). In contrast, there was no *gce-GAL4::p65* or *gce-T2-GAL4* expression in the wing, leg, eye, or genital imaginal discs of larvae (Fig. 5d–f) with any driver/reporter combination. To validate these results, we performed qPCR analyses with *Met*- and *gce*-specific primers, using cDNA libraries prepared from either pooled leg discs and wing discs, or from larval CNS’s which included associated leg discs. We employed two tissue-specific control genes to verify the integrity of the disc-specific sample: *Notum* was chosen for its enrichment in wing discs (ModEncode;^30^) while *bruchpilot* (*brp*), which marks presynaptic active zones in the CNS^31^, was used as a CNS-specific control gene. Consistent with our confocal data, we detected no *gce* transcript in wing/leg disc samples, while *Met* showed low expression in these tissues (Fig. 5g).

In contrast to the effects of exogenous JH on the differentiation of the adult abdomen derived from the histoblasts, structures derived from imaginal discs are not affected by JH treatment^27, 28^. We hypothesized that an absence (*gce*) or modest level (*Met*) of JHR expression in the discs might explain their JH insensitivity. To test this, we attempted to induce JH sensitivity, and presumably a reinduction of pupal cuticle, in wing discs, by supplying ectopic *gce*, *Met*, or both transgenes, using the c765-GAL4 driver, which expresses strongly in wing discs^32^, and rearing these larvae on food containing 1 ppm pyriproxyfen [a potent JHA for *Drosophila* ^6^]. However, none of these manipulations shifted the response of the wing discs to JHA (Fig. 5h and data not shown), suggesting that simply supplying additional JHR to imaginal discs is not sufficient to confer JH sensitivity to these tissues.

### Ring gland

**Figure 6:**
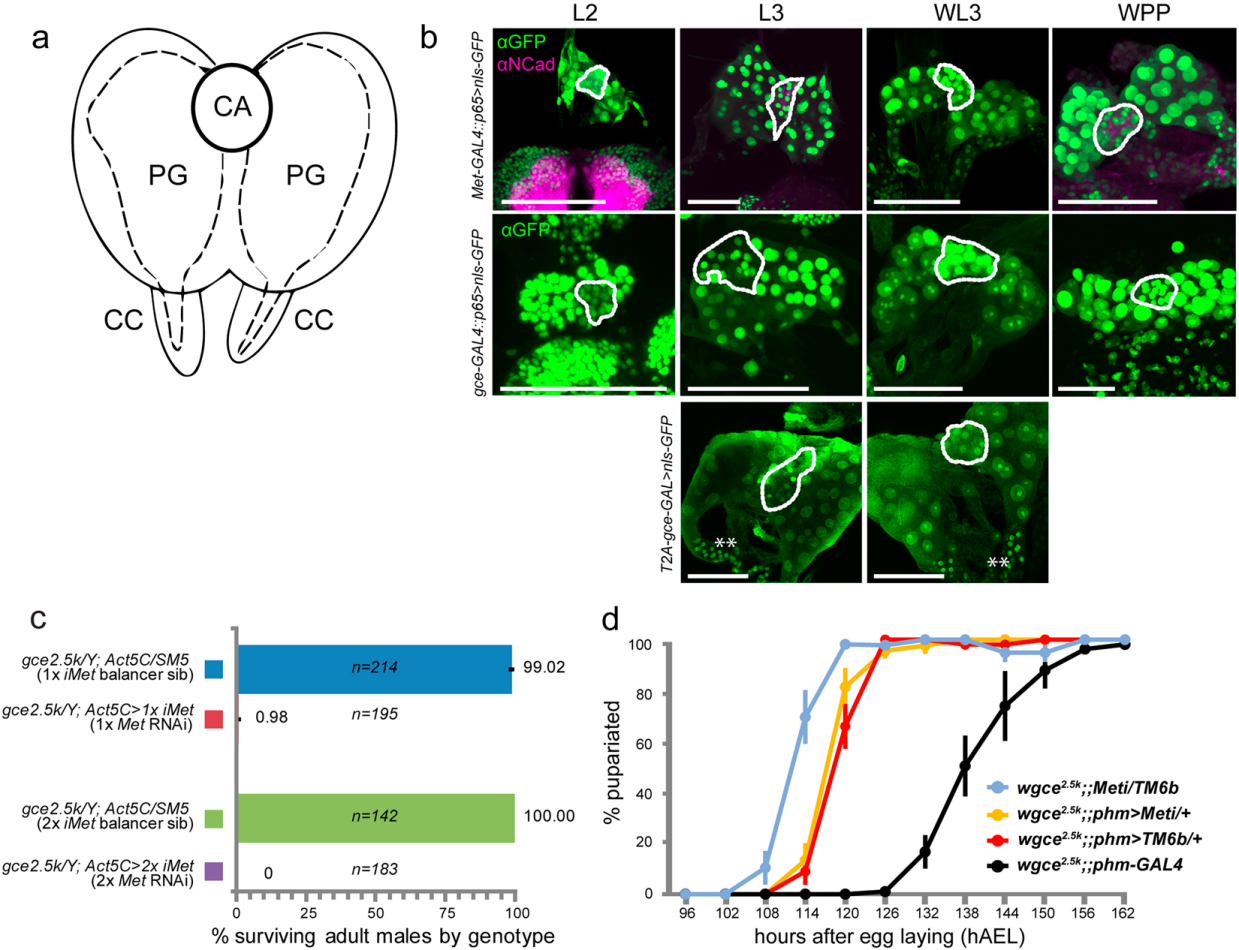
Presence of the JHRs in the ring gland. (**a**) Diagram of the endocrine tissues that comprise the larval ring gland, which includes the medial corpus allatum (CA), the paired lateral prothoracic glands (PG) that synthesize ecdysone, and the paired, basal corpora cardiaca (CC); (**b**) Confocal scans of the larval ring gland dissected from larvae expressing *nls::GFP* from either *Met*-*GAL4::p65*, *gce-GAL4::p65*, or *gce-T2A-GAL* drivers. Scans were taken from staged larvae from ~48 h after egg laying (AEL) (early L2 stage) to pupariation (WPP, ~120 h AEL). Where shown, N-cadherin labels the larval brain lobes and the CA. The position of the CA in each panel is indicated with a dotted line. (**c**) Global *Met* suppression was achieved by crossing *Act5C-GAL4* into *gce* mutants to drive *Met* RNAi (45854 or 45852). RNAi function was verified via phenocopying the effects of global *Met* and *gce* deletion, which results in pupal mortality. (**d**) Delay in time to pupariation in *gce* ^*2*.*5*k^; *phm* > *Met RNAi* larvae (n = 177) vs. control lines (*wgce;;Met-RNAi/Tm6b* parental stock, n = 170, *wgce* ^*2*.*5k*^;;*phm* > *TM6b* line, n = 162, and *wgce* ^*2*.*5*k^; *phm-GAL4* line, n = 335). Error bars: SEM. Scale bars, 50 µM. GFP (green or white), N-cadherin (magenta).

The ring gland of higher dipteran larvae contains the endocrine cells of the JH-producing corpus allatum (CA) and the paired, lateral prothoracic glands (PG), which synthesize and secrete ecdysone (Fig. 6a). As shown in Fig. 6b, we observed strong *Met* and *gce* expression in both the CA and PG during larval life; *gce-T2A-GAL4* > *nls-GFP* additionally showed strong expression in the corpus cardiacum (Fig. 6b, bottom row).

We sought to address the significance of JHR expression specifically in the PG, since this organ is considered a size-assessing tissue that regulates the time of metamorphosis^33^. We used RNAi to suppress *Met* expression specifically in the PG of *gce* ^*2*.*5k*^ mutants, effectively eliminating both receptors from the PG. *Met* RNAi was functionally validated since its expression driven by *Act5C*-*GAL4* in *gce* mutants produces the double mutant phenotype^12^. *gce* ^*2*.*5k*^; *Act5c* > *Met RNAi* larvae (either 1x *Met RNAi* in *gce* ^*2*.*5k*^ *; Act5C* > *45854* or 2x *Met RNAi* in *gce* ^*2*.*5k*^ *; Act5C* > *45854*, *45852*) showed nearly 100% pupal lethality (Fig. 6c; *n* = 195, 1x *Met RNAi*; *n* = 183, 2x *Met RNAi*), while balancer sibs showed approximately 100% survival to adulthood (*n* = 214, 1x *Met RNAi* cross; *n* = 142, 2x *Met RNAi*). *Met* suppression in the PG using *gce* ^*2*.*5k*^ *; phm* > *45852* was inconsequential for survival (*n* = 152/152).

Since an earlier study showed that Met suppression in the PG did not alter timing from the L2/L3 transition to pupariation^34^, we tested the hypothesis that *gce* expression may provide functional redundancy. We staged larvae in 6-hour egg collections and scored time to pupariation as the proportion of puparia to wandering larvae (Fig. 6d). *wgce* ^*2*.*5k*^;;*phm-GAL4* homozygotes were developmentally delayed beyond *wgce* ^*2*.*5k*^;;*phm* > *Met-RNAi* or *wgce* ^*2*.*5k*^;;*phm* > *TM6b* lines. The two *phm-GAL4* heterozygous lines showed nearly identical developmental timing profiles, but were slightly delayed relative to the *wgce* ^*2*.*5k*^;;*Met-RNAi*/*TM6b* parental stock. These data indicate that while *phm-GAL4* expression alone can induce a dose-dependent developmental phenotype, *Met* and *gce* suppression together in the PG does not alter developmental timing beyond the shift incurred by simply expressing *phm-GAL4*.

### Adult expression

Recent studies have revealed that reproductive behaviors are influenced by JH signaling, some of which require either Met or Gce^17^. The driver lines indicated that both JHRs were widely expressed in adults, with wide expressional overlap in many tissues. Here, we focus on expression in reproductive tissues and the adult CNS.

**Figure 7:**
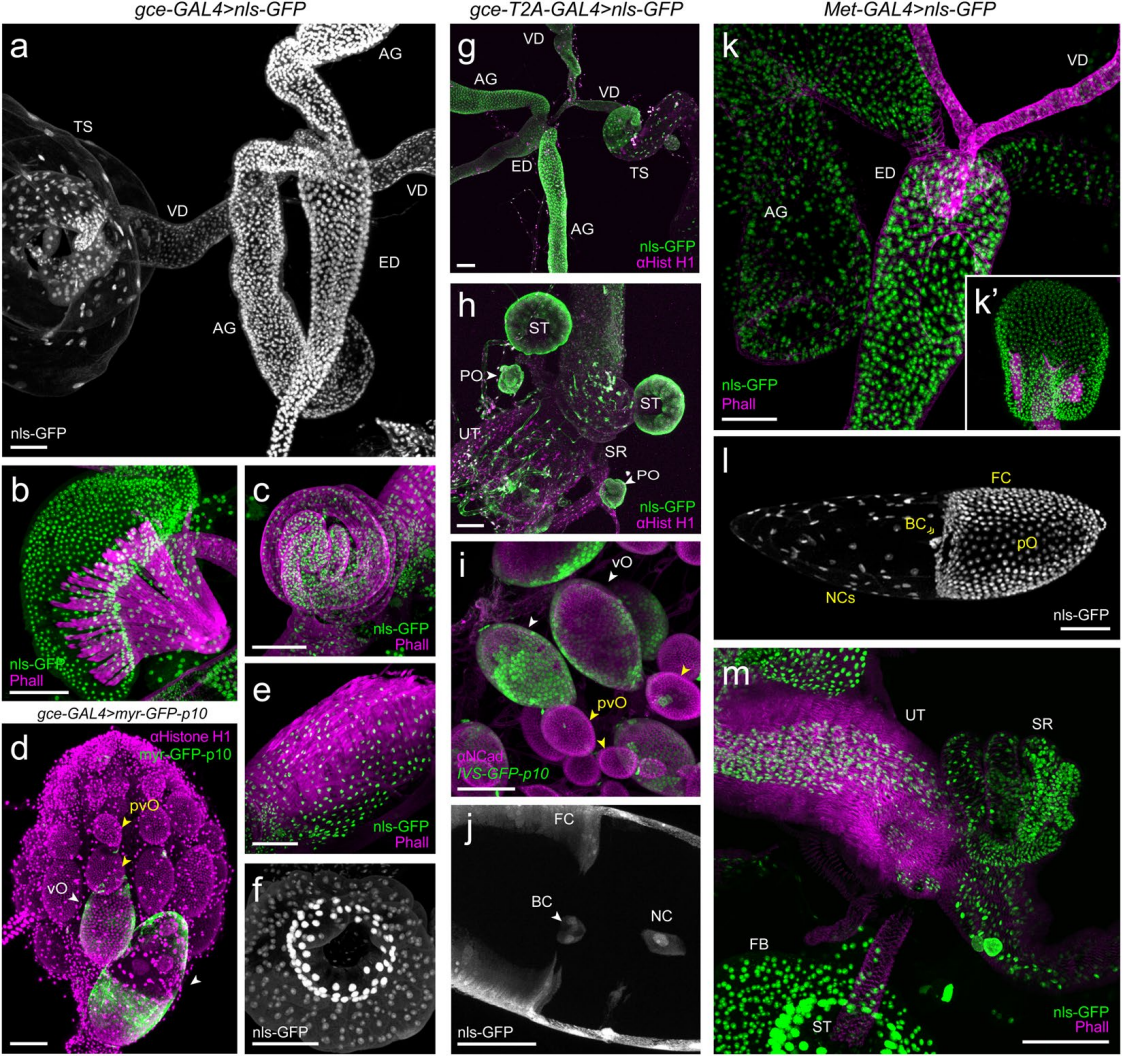
Confocal scans of reproductive tissues dissected from *gce-GAL4::p65* and *Met-GAL4::p65* lines driving *pStinger* (*nls::eGFP*) expression. **Left:** *gce-GAL4::p65* > *nls::eGFP* male reproductive tract expression: (**a**) male reproductive tract: ejaculatory duct (ED), accessory gland (AG), vas deferens (VD) and in the somatic cells of the testes (TS); (**b**) ejaculatory bulb (EB). *gce-GAL4::p65* > *nls::eGFP* female reproductive tract expression: (**c**) seminal receptacle (SR), the gross morphology of which is revealed with Phalloidin-568. (**d**) Previtellogenic oocytes (pvO; yellow arrowheads) showed no expression, while stage 8–10 oocytes (vO) undergoing the vitellogenic checkpoint, showed expression both in the follicle cells surrounding the primary oocyte and in the nurse cells; (**e**) uterus (common oviduct); (**f**) spermatheca (ST). **Center:** overall *gce-T2A-GAL4::p65* > *nls::eGFP* male (**g**) and female (**h**) reproductive tract expression, in which GFP expression is strong in paraovaria (PO) and spermathecae (ST). (**i**) GFP is absent from previtellogenic oocytes, and obvious in checkpoint-stage egg chambers; (**j**) close-up of the migrating border cells (BC) in a checkpoint-stage egg chamber. **Right:** *Met-GAL4::p65* > *nls::eGFP* expression was similar to *gce-GAL4::p65* > *nls::eGFP* in the male reproductive tract (**k**) and EB (**k’**); a vitellogenic oocyte shows strong GFP expression in the follicle cells surrounding the primary oocyte, in nurse cells, and border cells (BC). (**m**) The seminal receptacle (SR), uterus (UT) and spermathecae (ST) all showed intense GFP expression. GFP (green or white), Phalloidin-568 (magenta). All scale bars, 50 µM.

*Met* mutants suffer substantial reproductive deficiency, while *gce* mutants show only minor reductions in reproductive capacity^12^. Figure 7 provides an overview of *Met* and *gce* expression in male and female reproductive tissues as reported by the nuclear reporter UAS-*nls-GFP*. In males, both receptors show widespread expression; the ejaculatory duct and ejaculatory bulb, accessory glands and vas deferens all showed moderate to high level of *Met* and *gce* expression (Fig. 7a,b,g,k and k’). In females, both receptors showed strong expression in the spermathecae (Fig. 7f,h and m), regardless of mating status, as well as in the seminal receptacle (Fig. 7c,m), and uterus (Fig. 7e,h and m), but not in early stage oocytes (pvO in Fig. 7d,i, and data not shown). However, both receptors showed expression in the follicle cells of stage 8–10 oocytes, but *gce* UAS-*nls-GFP* expression in these cells was moderate compared to that of *Met* (Fig. 7j,l). We boosted expression with either cytoplasmic GFP or *myr-GFP-p10* to unequivocally identify *gce* in stage 8–10 oocytes (vO; Fig. 7d,i;). Reproductive tissues showed similar reporter expression for the *gce*-GAL4::p65 and *gce*-T2A-GAL4 drivers (Fig. 7a–j).

**Figure 8:**
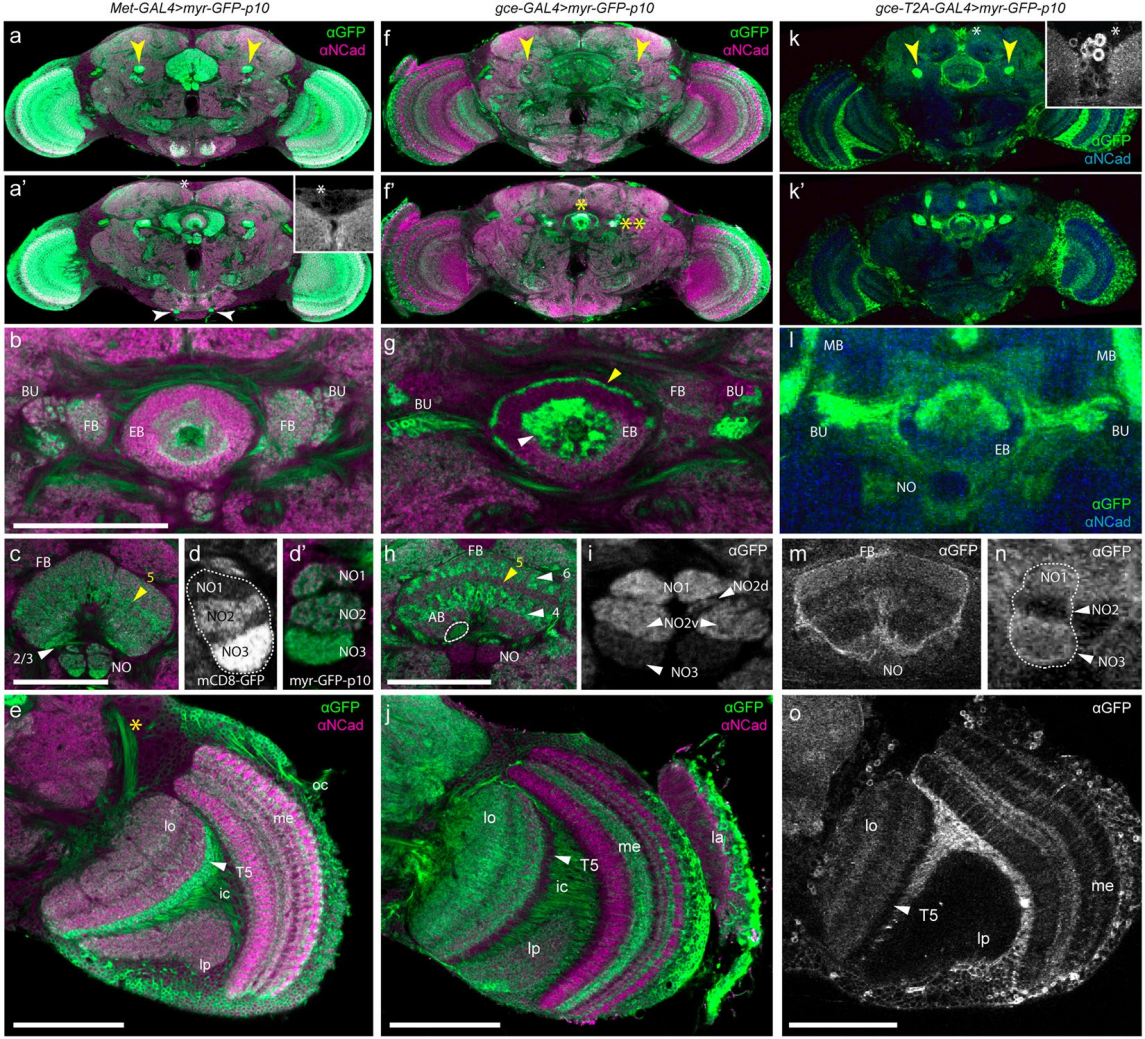
Confocal scans of JHR expression across the adult brain, with emphasis on central complex and optic lobe expression. Myr-GFP was used to visualize JHR expression in the nervous system. **Left:** Two z-slices showing the overall pattern of *Met* expression in the adult brain (**a**,**a’**). The mushroom bodies, which show strong Met expression, are indicated with yellow arrowheads in (**a**); pharyngeal nerves are indicated with white arrowheads in (**a’**); inset: minimal expression in a group of peptidergic cells (*). (**b**) *Met-GAL4::p65* > *myr-GFP-p10* expression across the compartments of the central complex, including the ellipsoid body (EB), fan-shaped body (FB), and bulb (BU). (**c**) Me*t* expression across the layers of the FB and nodulus (NO). Arrowheads indicate layers 2/3 (white) and 5 (yellow) of the FB. (**d**) mCD8-GFP expression and myr-GFP-p10 expression both show that *Met* is highly expressed in NO3 relative to other subcompartments. (**e**) *Met*-*GAL4::p65* **>** *myr-GFP-p10* expression across the optic lobe neuropils: lobula (lo), lobula plate (lp), medulla (me), showing strongest expression in the T5 arborizations (white arrowhead) that outline the periphery of the lobula. Yellow asterisk: anterior optical tract. **Center:** Two z-slices showing the overall pattern of *gce* expression in the adult brain. The mushroom bodies (yellow arrowheads) show only weak *myr-GFP* expression with the *gce*-GAL4::p65 BAC reagent (**f**,**f’**); high level *myr*-GFP expression evident in the ellipsoid body and bulb (**f’**) with one or two yellow asterisks, respectively; (**g**) inner (white arrowhead) and outer (yellow arrowhead) rings of the EB show intense *gce* expression; (**h**) *gce*-GAL4::p65 > *myr*-GFP-p10 expression across FB layers is strongest in layers 4 and 6 (white arrowheads) and comparatively weak in layer 5 (yellow arrowhead). Expression was also noted in the asymmetrical body (AB), outlined with a white dotted line. (**i**) NO3 (white arrowhead) shows minimal *GFP* expression, while NO1 and NO2 show moderate expression. (**j**) *gce* expression across the optic lobe neuropils, labeled as in **e**. *gce* was absent in the arbors of the T5 cells (white arrowhead). **Right:** Two z-slices showing the overall pattern of *gce-T2A-GAL4* > *myr-GFP* expression in the adult brain. Strong GFP expression is seen in the mushroom bodies (**k**) (yellow arrowheads) and in a subset of peptidergic cells (inset). Central complex expression is shown in (**l**–**n**); nodulus expression shows moderate to high GFP signal in NO1 and NO3 using *gce*-T2A-GAL4. (**o**) Optic lobe expression shows no GFP in the T5 cells (white arrowhead) or lobula plate (lp); medulla (me) expression is strong in some layers and weak in others. Scale bars, 50 µM. GFP (green or white), αN-cadherin (magenta).

To evaluate *Met*/*gce* expression in the brain, we employed a series of reporters specifically tailored for neuroanatomy^26, 35^. Overall, both receptors expressed broadly across the adult CNS, with each showing a unique and reproducible pattern. Indeed, the most striking examples of divergent JHR expression were observed across the various cell types of the adult CNS. For example, we observed distinct partitioning across compartments in the central complex and optic lobes (OL). An overview of both JHRs as reported with *myr-GFP-p10* is shown in Fig. 8(a,a’,f,f’,k and k’).

In the central complex, *Met* and *gce* expressed with variable intensity across the ring neurons of the ellipsoid body (EB), and either shared or occupied discrete layers of the fan-shaped body (FB). Across the layers of the FB, gce > *myr-GFP-p10* showed the most intense expression in layers 4 and 6, with comparatively weak expression in layer 5 (Fig. 8h), and also expressed in the asymmetrical body (AB), a unilateral structure associated with long term memory formation and retrieval^36^. *gce-T2A-GAL4* > *myr-GFP* exhibited a similar expression pattern, with weaker GFP signal evident in layers 2, 3, and 5 relative to other layers (Fig. 8m). *Met* > *myr-GFP-p10* showed global expression across the FB, with strongest expression shown in layers 1 and 5 (Fig. 8c). *Met* > *myr-GFP-p10* expression in the EB (Fig. 8b) was comparatively weaker than *gce* > *myr-GFP-p10* and *gce-T2A-GAL4* > *myr-GFP* (Fig. 8g,l), the latter of which was exceptionally strong relative to expression across the entire brain. The paired noduli (NO) displayed prominent dorso-ventral segregation of JHRs. *Met* > *myr-GFP-p10* showed highly enriched expression in the ventral NO3, with moderate expression in NO1 and NO2 (Fig. 8d’). In contrast, *gce-GAL4::p65* > *myr-GFP-p10* signal across the NO was moderate to strong in NO2 and NO1 (Fig. 8i), while *gce-T2A-GAL4* > *myr-GFP* showed minimal expression in NO2, with moderate expression in both NO1 and NO3 (Fig. 8n).

Given the staggering diversity of cell types in the OL, we focused on a handful of distinctive features that characterized the expression pattern of each receptor. *Met* > *myr-GFP-p10* and *gce* > *myr-GFP-p10* either coexpressed or expressed individually in the various cell types that constitute the layers of two of the major OL neuropils, the lobula and the medulla^37^, thus establishing a paralog-specific banding pattern in each neuropil (Fig. 8e,j). Some features of optic lobe expression were consistent between the BAC and knock-in *gce* reagents. None of the *gce* constructs drove reporter expression in the T4 or T5 layers of the medulla or lobula, respectively (Fig. 8j,o). There were also notable differences in the *gce* drivers. For instance, while BAC reagents showed moderate lobula plate expression (Fig. 8j), the T2A-GAL4 reagent did not drive myr-GFP expression in the lobula plate (Fig. 8o).

Expression of the JHRs in the medulla was complex with some layers showing expression of both receptors, other showing expression of neither, and still others with enhanced expression of Met or gce. Both *Met* > *myr-GFP-p10* and *gce* > *-myr-GFP-p10* as well as *gce-T2A-GAL4* expressed in axon bundles of cells crossing the inner chiasm (Fig. 8e,j and o). In the lobula, *Met* > *myrGFP* showed highest expression in layer 1 (Fig. 8e). By contrast, *gce* > *myr-GFP-p10* and *gce-T2A-GAL4* are lacking in layer 1 but are at high levels in the other layers of the lobula (Fig. 8j,o). Also, *Met* > *myr-GFP-p10* showed moderate expression in the medial-most layer 10 of the medulla occupied by arborizations of T4 cells, where *gce* was absent (Fig. 8j,o).

## Discussion

In this study we used a set of genetic tools to probe developmental expression of the duplicate *Drosophila* JH receptors *Met* and *gce*, by recapitulating their expression with fluorescent reporters. We have systematically surveyed larval and adult tissues, with emphasis on neuronal expression. Coexpression experiments in which the GAL4 and LexA *gce* reagents drove simultaneous GFP and tdTomato constructs demonstrated extensive expressional overlap between these reagents, but also illustrated some differences (Fig. 1e–g”). The *T2A-GAL4* knock-in and BAC reagents showed substantially divergent expression patterns in key CNS structures in both larvae and adults, likely a consequence of position effects and construct architecture, though there were shared features. Peripheral tissue expression was essentially the same between the T2A and BAC iterations of the *gce* constructs, indicating that at least in non-CNS tissues, BAC expression is a reliable proxy for endogenous *gce* expression. We obtained only a single *Met-GAL4::p65* BAC reagent; several efforts to produce a Met knock-in reagent were fruitless. Given the differences between BAC and knock-in data for *gce*, we propose that the *Met* BAC reagent faithfully recapitulates peripheral tissue expression but likely only approximates *Met* expression in the CNS, since we were unable to generate the “gold standard” genomic knock-in reagent for *Met*.

**Figure 9:**
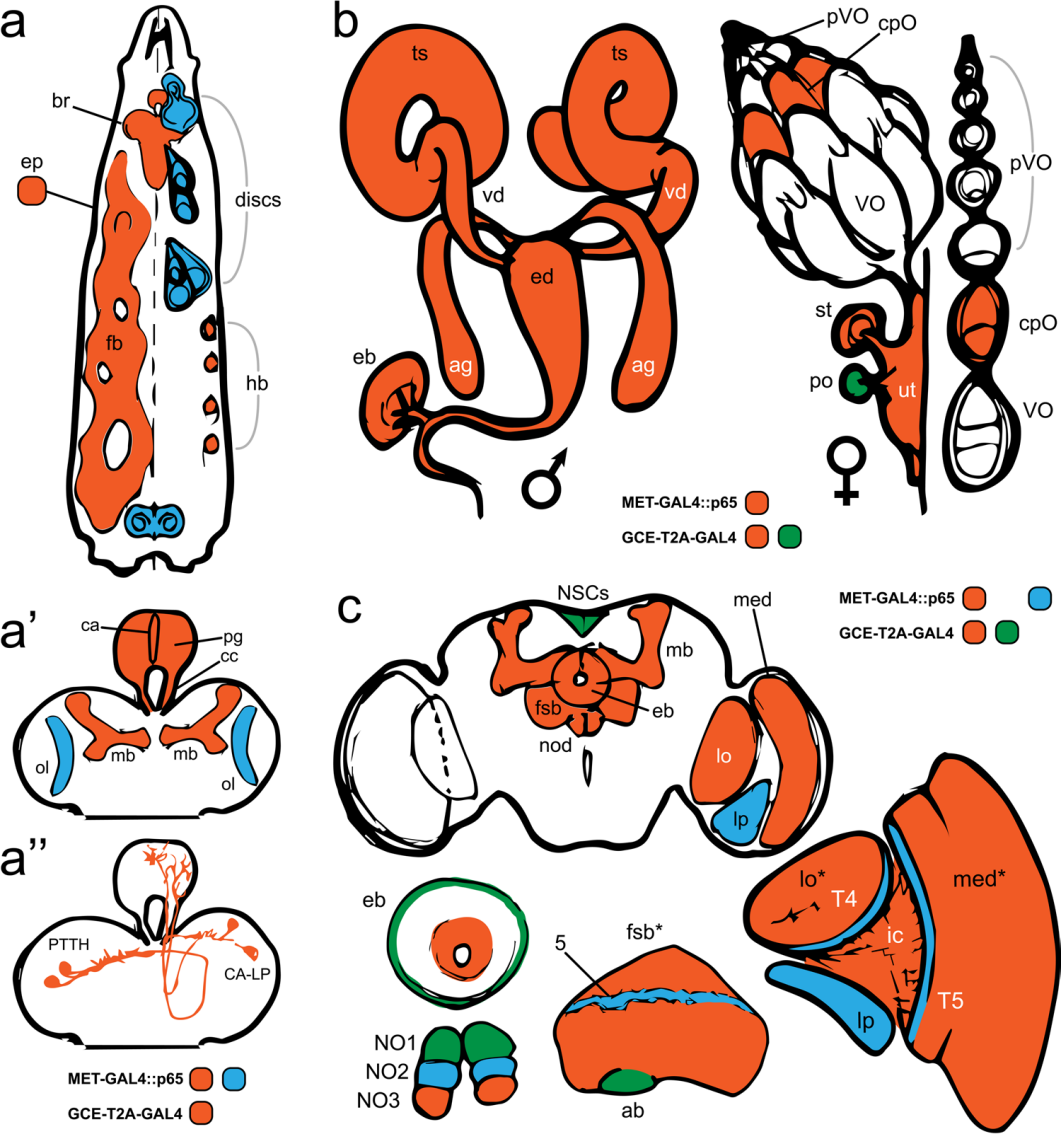
Summary of some major JH receptor expression features during larval and adult life. Boxes below each illustration match with the corresponding tissues in which reporter expression was observed, driven either by *Met-GAL4::p65* (blue), *gce-T2A-GAL4* (green), or both (red). (**a**) Overview of key larval expression: brain (br), epidermis (ep), discs, fat body (fb), histoblast nests (hb). (**a’**) Expression in the larval CNS and ring gland; corpus allatum (ca), prothoracic gland (pg), corpora cardiaca (cc), mushroom bodies (mb), developing optic lobe (ol). (**a”**) JHR expression in the PTTH and CA-LP cells, which innervate the PG and CA, respectively. (**b**) Male (left) and female (right) reproductive expression: testis (ts), vas deferens (vd), ejaculatory duct (ed), accessory gland (ag), ejaculatory bulb (eb), previtellogenic oocyte (pVO), checkpoint oocyte (cpO), vitellogenic oocyte (VO), spermatheca (st), paraovarium (po), uterus (ut). (**c**) Key expression features in the adult brain: neurosecretory cells (NSCs), mushroom bodies (mb), ellipsoid body (eb), nodulus (nod; below shows individual nodulus subunits NO1, NO2, NO3), fan-shaped body (fsb*; detail below shows *Met-GAL4::p65* expression in layer 5 (5) and *gce-GAL4::p65* expression in the asymmetrical body (ab)), lobula (lo), lobula plate (lp), inner chiasm (ic), medulla (med*); detail of optic lobe expression showing Met-GAL4::p65-specific expression in the T4 and T5 cells and lp. *Indicates paralog-specific expression across various cell types of the indicated neuropil.

Overall, our confocal data are consistent with characterized aspects of tissue-specific JH action and therefore represent a working foundation for future studies regarding JH action at the cellular level in *Drosophila*. An overview of key expression features in the larva is given in Fig. 9a–a”. JHR redundancy during larval life^12^ is supported by their near global coexpression. A key exception to this finding was that *gce* but not *Met* was conspicuously absent from the imaginal discs (Fig. 5a,b and c). Following JHA challenge, disc-derived structures develop normally in *Drosophila* ^6,27, 28^. In contrast, exogenous JHA reprograms the abdominal histoblasts by maintaining the expression of the pupal specifier *Broad*, promoting a second pupal, rather than adult, epidermis^38^. We demonstrate here that *Met* and *gce* coexpress in histoblasts during the window of JH sensitivity^28^, following which their expression declines, with *gce* levels declining followed by *Met*, beginning at 9 h APF (Fig. 4). Under the rationale that JH sensitivity requires both receptors, we created lines in which *Met*, *gce*, or both were ectopically expressed specifically in the wing disc. However, following dietary JH treatment, wing development in both experimental and balancer flies was normal (Fig. 5h), indicating that ectopic JHR expression was not sufficient to confer JH sensitivity to this tissue. To address the possibility that another component of the JH receptor machinery was absent in wing discs, we labeled the discs with antibodies against the JH-dependent Met binding partner Taiman (Tai)^39, 40^. Tai was observed in discs in late wandering larvae, before the onset of pupariation (Fig. S1). Therefore, the canonical JH signaling pathway is present. Perhaps unidentified inhibitory factors block JH action in wing discs or downstream components are missing.

Removing JH signaling in *Drosophila* larvae, through genetic ablation of the CA [allatectomy (CAX); 13, 14] or by suppressing *Met*/*gce* function^12^, cannot prevent the onset of metamorphosis, but does alter developmental timing in the third instar. During this instar, the PG establishes the timing of pupariation by assessing larval size to determine whether the critical weight checkpoint, which corresponds to the time after which starvation cannot prevent metamorphosis, has been attained^33^. By suppressing Met expression specifically in the PG, Mirth *et al*.^34^ demonstrated that this treatment is insufficient to alter time to pupariation relative to *phm*/+ control larvae. In this study we found Met and Gce were coexpressed in the PG during larval development (Fig. 6b), suggesting the possibility that Mirth *et al*.^34^ failed to override functional redundancy between JHRs in the PG. To address this, we performed the same manipulation in *gce* mutants, eliminating PG expression of both JHRs. There was no difference in pupariation time between the Met RNAi larvae and the Tm6b balancer sibs although both were delayed slightly in comparison to the parental stock, indicating that the *phm*-*GAL4* construct itself can influence the time to pupariation. Our finding that the *gce* mutant with two copies of the *phm-GAL4* construct showed a large delay in pupariation time indicates that this delay is dose-dependent. Thus, the *phm-GAL4* construct alone is sufficient to shift time to pupariation, a phenotype that is consistent with perturbed ecdysone synthesis or PG function^41^. When we attempted to create a *wvMet* ^*27*^;;*phm*/*TM6b* line, the stock was fragile and showed extensive preadult mortality, indicating that *Met* flies are sensitive to PG manipulation, and that expression of GAL4 alone in the PG has developmental consequences.

Reciprocal biosynthetic regulation between JH and ecdysone pathways has been demonstrated in several species, though the mechanisms segregate along taxonomic lines. In the hemimetabolous *Diploptera punctata*, expression of the ecdysone receptor proteins EcR and RXR (USP) coincides with an elevation in JH titer in 5-day old adult females; suppressing these genes lowers ecdysteroid titer and elevates JH biosynthesis in 6-day old females^42^. In holometabolous insects, 20E has an evolutionarily-conserved allatotropic role. JH biosynthesis is irreversibly stimulated in cultured *Aedes aegypti* CA following 20-hydroxyecdysone (20E) addition at a concentration of 10^−6^ M^43^. Fourth instar *Bombyx mori* CC-CA complexes likewise increased JH biosynthesis when cultured with 20E in a narrow concentration range, and this JH synthesis profile was concomitant with the elevation and subsequent decline in the transcript abundance of two JH biosynthetic enzymes^44^. In both *A*. *aegypti* ^43^ and *B*. *mori* ^44^, 20E overrides an inhibitory factor from the brain that prevents JH synthesis at a developmentally inappropriate time.

In *Drosophila* such a factor(s) may be a product of the CA-LP1/2 neurons that innervate the CA^45^, and which according to the present study, show JHR expression in WL3 larvae. Further, JH receptor expression in CA/PG cells, as well as in CA-LP and PTTH neurons suggests a potential role in coordinating hormone biosynthesis. For instance, by responding to changes in hormone titer, JHRs may participate in a feedback mechanism through which one or both receptors monitor the rate of biosynthesis or release of JH, ecdysone, or both. Experiments in other insects have shown that JH can inhibit PTTH release to indirectly suppress ecdysone production^46, 47^, or act directly on the PG to suppress ecdysteroid synthesis^48^. Met and Gce both express in the PTTH cells (Fig. 3k,l) and PG (Fig. 6b) during the third instar. The coexpression of JH and ecdysone receptor machinery in the endocrine cells and associated neurons supports these tissues as critical sites for the convergence of signaling pathways, and the sensitivity of *wvMet* ^*27*^;;*phm*/*TM6b* flies to PG manipulation suggests that Met may be the key player in integrating these signals.

In adult flies, JH-Met regulates reproductive physiology in both sexes^7, 49^. *Met* mutants produce and oviposit fewer vitellogenic oocytes than wild type flies^7^, while flies lacking *gce* exhibit considerably milder reproductive consequences^12^. We observed widespread *Met* and *gce* coexpression in both male and female reproductive tracts (Figs 7 and 9b).

In *Drosophila* oogenesis, JH is necessary for entry into the vitellogenic phase^50^. The vitellogenic checkpoint occurs in stage 9^51^ oocytes where JH is required for both yolk protein synthesis by the follicle cells and yolk protein uptake from the hemolymph^50^. It also suppresses 20E-induced apoptosis of the oocyte at stages 8–9 when the females are starved^52, 53^. This apoptosis is mediated by the ecdysone-induced E75A^53^. Both *Met* and *gce* are expressed in the follicle cells surrounding the primary oocyte in stage 8–10 egg chambers, which corresponds with the vitellogenic checkpoint (Fig. 7h,m and n). Met and gce in stage 8 and 9 follicle cells may mediate the action of JH, 20E, or both to oppose apoptosis of egg chambers. The comparatively severe oogenesis phenotype exhibited by *Met* mutants argues for a scenario under which Met-JH suppresses ecdysone-driven apoptosis, possibly by downregulating the apoptosis inducer E75A, which is a JH and 20E responsive gene, and a demonstrated transcriptional target of JH-Gce in cell culture^54^. If *gce* regulates E75A expression in check-point stage egg chambers, *gce* overexpression, rather than loss of function, might be expected to promote apoptosis in egg chambers.

Virtually nothing is known about *Met* and *gce* function in the brain, but a handful of studies have addressed the more general issue of JH action on nervous system development. Widespread and dramatic neurological damage results from a lethal dose of the JH analog methoprene in OR-C wildtype flies, including fusion of the subesophageal ganglion with the thoracicoabdominal ganglion, an incomplete fusion of the optic lobe neuropils with the central brain, and disorganization and degeneration of the optic lobe, particularly in the chiasma^22^. Riddiford *et al*.^14^ describe a gross malformation of the lobula in *Met* ^*27*^ null mutants, evident as finger-like projections that encroach laterally into the inner chiasm, attributed partially to premature EcR B1 expression. *gce* mutants do not display this phenotype^55^. Accordingly, *gce* expression in the developing optic lobe of WL3 larvae is sparse (Fig. 3b,e), and the T5 cells that arborize along the margin of the lobula likewise show *Met* but not *gce* expression (Fig. 8e vs. 8j,o), suggesting a potential maintenance function for Met-JH in the morphological integrity of the developing lobula.

The neuroanatomical atlas of JHR expression presented here will allow targeted studies regarding JH effects on behavior. Key expression in the adult CNS is summarized in Fig. 9c. In *Drosophila*, JH application or the pharmacological suppression of JH synthesis via precocene application in the adult induces changes in basic locomotor behaviors and courtship^56^. The central complex is a major locomotor control center in the brain^57^ and shows strong *Met* and *gce* expression in distinct cell types (Fig. 8). A recent study by Wolff *et al*.^58^ provides an exquisite reconstruction of the central complex at single-cell resolution and identifies a collection of GAL4 lines that grant genetic access to these neurons. Such collections will facilitate future research regarding JHR action in coordinating CNS development and function. A general strategy will require defining JHR-carrying, hormone-responsive neurons via co-expression of *Met*/*Gce*-GAL4 lines with LexA driver lines that express in characterized sets of neurons. We envision that the genetic tools presented herein will facilitate such research and help elucidate how developmental hormones interact to direct behavior.

## Materials and Methods

### Bacterial Artificial Chromosome (BAC) generation for making driver lines

BAC recombineering was performed in SW102 cells^59^ using a kanamycin/streptomycin positive/negative-selection cassette modified from *pSK* + *-RpsL-kana* ^60^. Landing-site cassettes were created by ligating ~500-base-pair GAL4 or LexA 5′ and 3′ homology arms amplified from *pBPGUw* ^25^ and *pBPnlsLexA::p65Uw* ^26^, creating *pGKU* and *pLKU*. Landing-site cassettes were amplified from *pGKU* and *pLKU* using primers that added 50 bases of 5′ and 3′ *Met* and *gce* homology to the GAL4/LexA-flanked selectable markers. These cassettes were recombined into the *Met* or *gce* BACs (CH321-28O05 and CH321-09E09:^61^) (obtained from Children’s Hospital Oakland Research Institute, Oakland, CA), and recombinants were selected on kanamycin medium. Putative clones were analyzed by colony PCR to ensure that proper recombination had occurred. Full-length *GAL4*, *GAL4::p65*, or *NLS::LexA::p65* transgene cassettes were recombined into these modified BACs making use of the 500-bp homology arms added earlier. Potential recombinants were identified by faster growth on streptomycin medium. Clones were analyzed by colony PCR, and putative recombinant BACs were sequenced at the recombined regions. Correct BACs were isolated and electroporated into EPI300 cells (Epicentre) and EPI300 clones were shipped to Genetic Services, Inc. (Cambridge, MA), as agar stabs for preparation and injection into *y w nos phi C3*-*nls* #12; attP40; VK00033 flies (hereafter referred to as the injection line). Integrant flies were analyzed by PCR to ensure proper integration of the transgene.

### Fly lines

Reporter lines used in this study include the membrane-bound GFP reporters *10xUAS-IVS-mCD8-GFP* (pJFRC2; attP2), *10xUAS-IVS-myr-GFP* (pJFRC12; attP2), *10xUAS-IVS-GFP-p10* (pJFRC28; attP2), *10xUAS-IVS-myr-GFP-p10* (pJFRC29; attP2;^26^), *13xLexAop2-IVS-myr-GFP* (pJFRC19; attP2;^26^), *LexAop2-IVS-myr-tdTomato-p10* (attP40), and the nuclear GFP reporter *UAS-nls-eGFP* (pStinger;^62^). Other *Drosophila* lines used in this study include: *wgce* ^*2*.*5k*^ ^12^, *phm-GAL4* ^33^, Met RNAi lines (BDSC #v45852 (*w* ^*1118*^; *P{GD4384}v45852*) and #v45854 (*w* ^*1118*^; *P{GD4384}v45854*)), *Act5C-GAL4* (BDSC# 4414, *P*{*w* ^+*mC*^ = *Act5C-GAL4*}*25FO1*/*CyO*, *y* ^+^), *w*; *UAS-Met* (attP40), and *w*; *UAS-gce* (attP2). The *PTTH*-*HA* line was a gift from Dr. Michael O’Connor.

### Quantitative PCR

Quantitative real-time PCR was performed on a LightCycler 480 (Roche Molecular Diagnostics, Basel, Switzerland) using SYBR Green detection. Data were analyzed using the ΔΔCT method.

### Gce-T2A-GAL4 construction

To knock-in the T2A-Gal4 coding sequence immediately preceding the *gce* stop codon^63, 64^, 5′ and 3′ homology arms approximately 3 kb in length were amplified with the following primers: gce_55_AgeI: TACGACCGGTCCACATTATTCTGACATTTTAGTCTGAG; gce_53StuI: AAGGCCTGTCCTGGTCGTCCTCCTG; gce_35PmeI: TACGGTTTAAACTAGAGTGATGGAGAGCGCC; gce_33MluI: TACGACGCGTACACCTTGCCAAACAATGACAC BAC from clone BACR48D17, and cloned into pTL1^65^. T2A-Gal4 was generated by cloning Gal4 from pBPGAL4.2Uw-2^26^ into pTL1 (StuI/SacI) using a forward primer containing the T2A coding sequence. Donor DNA was integrated at attP40 to create the corresponding transgenic fly, which was then mated with+*/Y*, *hs-hid; 5X-ri6TS-Rac1* ^*V12*^ *; hs-FLP*, *hs-I-SceI/TM3*, *Sb* ^66^. The progeny were heat shocked (37 °C for 1 hour) twice, once during the second instar (L2) and once during the mid-third instar (L3) to promote donor release. Females, *Donor*/*5X-ri6TS-Rac1* ^*V12*^ *; hs-FLP*, *hs-I-SceI*/+, were crossed to *nSyb-LexA::p65* for lethality selection. Surviving candidates were subjected to chromosomal mapping and genomic PCR confirmation. An identical approach was taken to construct a *Met-*T2A-GAL4 reagent, but we were unsuccessful in generating transformant lines.

### JH treatment

Five µg pyriproxifen (Sumitomo Chemical Company, Osaka, Japan) (a JHA) in 50 µl 95% ethanol was mixed into 5 ml molten standard molasses/cornmeal/yeast diet after cooling to 60 °C to make a 1 ppm pyriproxifen diet. Control food contained 50 µl of 95% ethanol only. Flies were monitored for pharate adult mortality, the typical wild type response to high doses of JHAs^6^.

### Genomic rescue

Native BACs (CH321-28O05 and CH321-09E09^61^; called “*Native Gce*” or “*Native Met*”) lacking a driver cassette were used to transform the same injection line used for driver line construction. Transformants were homozygosed in a *w* ^*1118*^ background. Restoration of functional JH signaling was assayed by scoring male survival from crosses with *Met* ^*27*^, *gce* ^*2*.*5k*^/*FM7c* females.

### Immunocytochemistry

Animals were dissected in phosphate-buffered saline (PBS; pH 7.4, from 10x biology grade PBS, Corning 46-013-CM) and fixed in 4% formaldehyde in PBS for at least 2 hours. Following three washes with PBS +1% TritonX (1% PBST), tissues were blocked in normal donkey serum at a final concentration of 1:50 for at least 2 hours. Primary antibodies were supplied in the following concentrations: chicken anti-GFP monoclonal at 1:1000; mouse anti-Histone H1 at 1:1000; rat anti-N-cadherin (DNEX-8) at 1:20; mouse anti-Fas2 (1D4) at 1:50; rabbit anti-dsRed at 1:500. Following incubation at 4 °C for at least one night, tissues were washed three times in 1% PBST and secondary antibodies in 1% PBST were added at the following concentrations: goat anti-chicken AlexaFluor 488 at 1:1000, donkey anti-mouse Cy5 at 1:1000, donkey-anti rabbit AlexaFluor 594 at 1:1000, and donkey anti-rat AlexaFluor 649 at 1:1000. Following overnight incubation at 4 °C for at least one night, tissues were washed three times in 1% PBST, placed on poly-L-lysine coated cover slips, dehydrated and cleared through an ethanol/xylene series, mounted in DPX mounting medium, and visualized by confocal microscopy on a Zeiss 510 LSM^67^.

## Electronic supplementary material

Figure S1
