# Supplementary material for: Genetic tools to study juvenile hormone action in Drosophila
Source: Sci Rep. 2017 May 18;7:2132. doi: 10.1038/s41598-017-02264-4 (PMC5437021; doi:10.1038/s41598-017-02264-4)
Supplement: Supplementary file 1 — Figure S1 [file 41598_2017_2264_MOESM1_ESM.pdf]

Genetic tools to study Juvenile Hormone Action in *Drosophila*: Supplementary data

Baumann, A.A.[1,2], Texada, M.J.[1], Chen, H.M.[1], Etheredge, J.N.[1], Miller, D.L.[1,3], Picard, S.[1], Warner, R.[1], Truman, J.W.[1,4], Riddiford, L.M.[1,4]

[1]Howard Hughes Medical Institute, Janelia Research Campus, Ashburn, VA 21047
[2] Present address: University of Tennessee, College of Veterinary Medicine, Knoxville, TN 37996
[3] Present address: National Institute of Neurological Disease and Stroke, NIH, Bethesda, MD 20892
[4] Present address: Friday Harbor Laboratories, Friday Harbor, WA 98250

Figure S1. GFP and anti-Tai antibody staining in imaginal tissues. *esg-GAL4* was used to vizualise coexpression of GFP and Tai in WL3 imaginal discs and histoblast nests.

(a) Tai (red) and myr-GFP expression in the leg disc. **(b and b')** Tai and myr-GFP expression in histoblasts nests. Red: anti-Taiman. Scale bars, 50 µM.

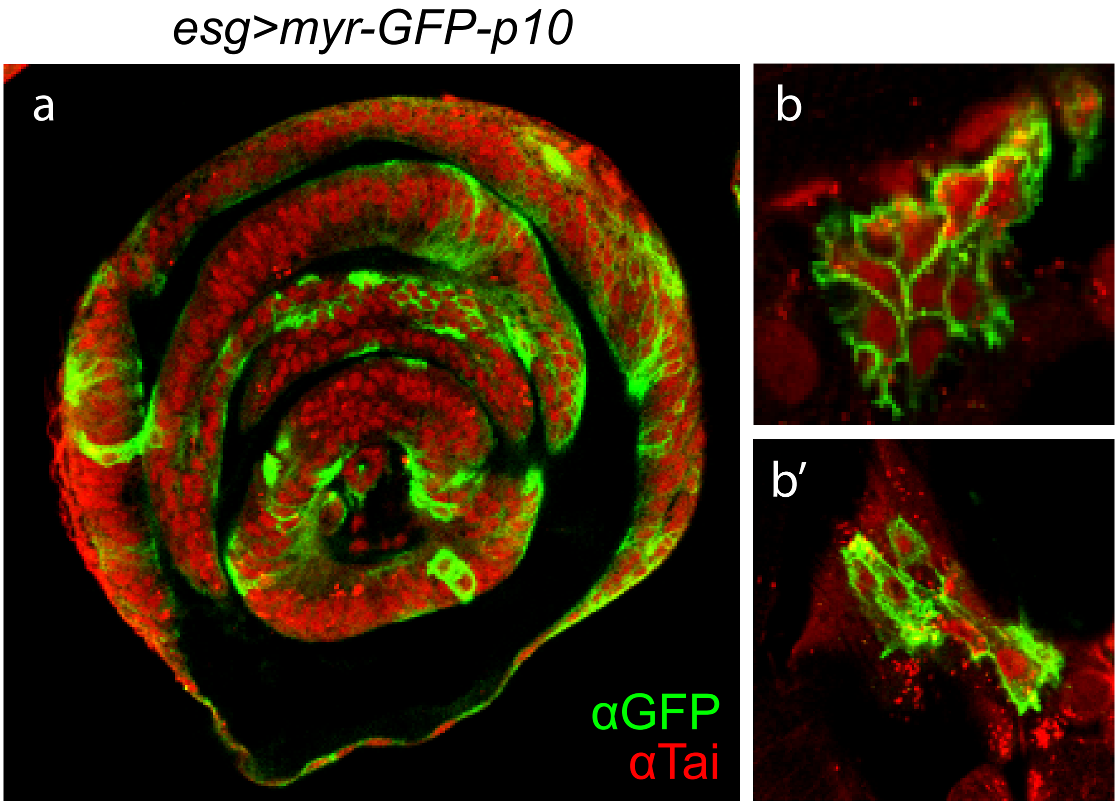
esg>myr-GFP-p10
a
b
b′
αGFP
αTai
